# Why are so few patients rating their physicians on German physician rating websites? A qualitative study

**DOI:** 10.1186/s12913-018-3492-0

**Published:** 2018-08-29

**Authors:** Stuart McLennan, Daniel Strech, Hannes Kahrass

**Affiliations:** 1 Institute for Biomedical Ethics, University of Basel, Bernoullistrasse 28, 4056 Basel, Switzerland; 2 Berlin Institute of Health, Quest Center for Transforming Biomedical Research, Berlin, Germany; 3 Institute for History, Ethics and Philosophy of Medicine, Hannover Medical School, Hannover, Germany

**Keywords:** Physician rating websites, Patient satisfaction

## Abstract

**Background:**

Physician rating websites (PRWs) allow patients to rate, comment and discuss physicians’ quality online as a source of information for others searching for a physician. It is generally assumed that PRWs will only be helpful for users, and fair for the rated, if there are a high number of ratings. However, the number of ratings on PRWs remains low internationally and there is currently a lack of research examining the reasons why patients are not rating their physicians. The aim of this study is to therefore identify the spectrum of factors influencing people’s willingness to rate their physician on PRWs.

**Methods:**

A mailed cross-sectional survey sent to a random sample from 4 North German cities between April and July 2016. Fifty participants who had previously used PRWs but not rated a physician provided reasons for why that had not rated a physician in a free text response. Semi-structured qualitative telephone interviews were then conducted with 22 interested participants to explore factors influencing their willingness to rate their physician on PRWs in more detail.

**Results:**

Participants identified a total of 21 distinct incentives and disincentives for rating physicians on PRWs, which could be further categorised under the headings: user-specific, PRW-specific and physician-specific. Two key overarching groups of factors emerged: (1) factors concerning the physician-patient relationship, and (2) factors issues regarding technical aspects of PRWs.

**Conclusion:**

These findings will be helpful in guiding future research and health policy initiatives to increase the usefulness and fairness of PRWs.

## Background

The past decades have seen greater transparency around healthcare quality, with an increasing number of activities publically reporting quality information with the aims of supporting patient decision-making and quality improvement [1, 2]. Physicians rating websites (PRWs) represent a bottom-up public reporting approach with their emphasis on user generated content (Web 2.0), by allowing patients to rate and comment on physicians’ quality online as a form of electronic word of mouth [3–10].

While a number of empirical studies have indicated that PRWs are having some success in influencing patient decision-making and health care quality [11, 12], a number of short comings of PRWs have also been identified in the literature [13]. One of the key concerns identified about PRWs is the low number of physician ratings that are overwhelmingly positive, which has called into question the representativeness, validity and usefulness of information on PRWs [13]. A recent study which examined the frequency of ratings and evaluation tendencies on a selection of German PRWs and compared this with 2010 data, indicates that the number of ratings per physicians remains low and very positive [14].

There has been to date, however, only limited research examining the reasons influencing people’s willingness to use of PRWs and to rate their physicians. At the most basic level, the use of PRWs requires people to first be aware of them [15], and it has been suggested that one reason for the low usage of PRWs might be that patients are still unaware of these websites [5]. However, recent surveys in the U.S. and Germany suggest that a lack of PRWs awareness is no longer a key barrier to PRWs usage in these countries. Two U.S. studies published in 2014 by Hanauer et al., found that while 74% of parents and 65% of adults in a nationally representative sample of the U.S. population were aware of PRWs [16, 17]. While a recent study in Germany by McLennan et al. reported that 75% of respondents were aware of PRWs [18]. Furthermore, while the level of PRWs awareness reported by these studies were higher, the level of PRWs usage was found to be comparable with previous studies [9, 11]. This suggests that even if awareness of PRWs increases, there may be other factors behind the low level of PRWs usage.

The finding of an online survey of 1006 randomly selected German patients conducted in 2012 by Terlutter et al. suggest that one important factor influencing PRWs usage if the personal characteristics of users. It was found that people with higher education, poorer health status, higher digital literacy, lower importance of family and pharmacist for health-related information, higher trust in information on PRWs, and higher appraisal of usefulness of PRWs, were significant predictors for PRWs usage [9].

While we are not aware of previous research directly examining the factors influencing people’s willingness to rate their physician on PRWs, there have been several studies that have reported important insights. In a study by Patel et al., which examined patients´ views regarding rating general practitioners on PRWs within the context of other feedback methods available in England, it was reported that participants would not leave feedback on PRWs because of accessibility issues; privacy and security concerns; and because they felt feedback left on a website may be ignored [19]. In a survey of a nationally representative sample of the U.S. population, Hanauer et al. also asked participants to consider the implications of leaving negative comments about a physician; participants reported being concerned that their identity could be disclosed, and that the physicians may take action against them for leaving negative comments [17]. Finally, recent studies by Rothenfluh et al. indicate that a number of patients perceive that they have an inability to properly evaluate the skills and abilities of physicians [20, 21].

Further research is needed to identify the factors influencing people’s willingness to rate their physician on PRWs to assist efforts to increase the number of ratings on PRWs and, consequently, improve the fairness and practical importance of PRWs. The aim of this study was to therefore identify the spectrum of factors influencing people’s willingness to rate their physicians on PRWs.

## Methods

The study was approved by Hannover Medical School’s Research Ethics Committee on 12 January 2016. All participants signed an informed consent form. The methods of the study are presented in accordance with the “Consolidated criteria for reporting qualitative research” (COREQ) [22].

### Research team and reflexivity

Interviews were conducted by H.K., a male Post Doc in biomedical ethics who has previous training and experience in qualitative research, and S.S. (see acknowledgement action), a male medical student who has had moderate previous experience in qualitative research. No relationship was established between the interviewers and the participants prior to the study and participants received limited information about the interviewers. There was no hierarchical relationship between the interviewers and the study participants. D.S. and S.M. both have longstanding experience with qualitative studies.

### Study design

The theoretical framework employed in this study was conventional content analysis [23]. Participants were initially recruited via an anonymous survey conducted between April and July 2016 [18]. Surveys were mailed to 1600 residents of four cities in north Germany (Nordhorn, Hildesheim, Bremen or Hamburg). A total of 280 completed surveys were returned, corresponding to an 18% response rate (280/1542). Participants who had previously used PRWs but not rated a physician were asked to provide reasons for why that had not rated a physician in a free text response. A total of 50 free text responses were received.

Telephone interviews were then conducted with interested participants (who may or may not have provided a free text response) to explore factors influencing people’s willingness to rate their physicians on PRWs in more detail. Possible interview partners were identified via survey consent forms, where survey participants´ were also asked if they would be willing to be interviewed for the project, along contact details and preferred time for an interview. Purposive sampling was used to focus primarily on recruiting participants who had previously used PRWs but had not rated a physician. In addition, participants who had rated a physician on PRWs were also recruited. Telephone interviews were held between April 2016 and February 2017. Only the participant and the researcher were present during the interview.

The initial survey sample consisted of 280 participants. As previously reported [18], 16% of respondents were aged 30 years and less (44/280), 29% were aged 30 to 50 years (82/280), 39% or respondents were aged 50 to 70 years (108/280), and 16% were aged 70 years and above (46/280). Fifty-five percent of respondents were female (154/280), 59% were married or in a civil partnership (165/280), 78% had never been employed in healthcare (218/278), 81% had public health insurance (227/279), 35% suffered a chronic illness (98/279), 30% had changed their place of residence in the last 10 years 1–2 times (83/279), and 10% had changed three or more times (28/279). Analysis of these sociodemographic factors found that only participants’ gender (higher for females than for males) and marital status (higher if married or in a civil partnership than if not) on awareness of PRWs and responders’ age on use of PRWs (higher for younger) had a relevant impact. No sociodemographic factors were found to predict a participant previously rating a physician on a PRW [18]. Of the 22 participants involved in the telephone interviews, 55% (12/22) were female, with a mean age of 56 years (range 23 to 84 years).

Interviews were conducted using a researcher developed semi-structured interview guide. Participants were asked to respond to the following four open-ended questions: *1) What is your general impression of physician rating websites? 2) How do you use physician rating websites? 3) Why have you not yet rated a physician on a physician rating websites? and 4) Under what circumstances could you imagine rating a physician on a physician rating websites?* If the participant had already rated a physician, questions 3 and 4 were replaced with the following questions: *3) What were your motivations and experiences with rating a physician on a physician rating websites? and 4) Will you give further physician ratings in the future and what could be improved?* Based on the first 2 interviews which did not show any problems, it was decided that no further piloting or adaptation of the interview guide was necessary. No repeat interviews were carried out. Interviews were audio recorded and transcribed verbatim. Interviews lasted an average of 11 min (ranging from 4:14 min to 28:27 min). After 22 interviews the question about data saturation arose and discussed by the research team. It was concluded that saturation was reached in the content and attitudes expressed by the participants on the main themes. Transcriptions of the interviews were not returned to the participants.

### Analysis and findings

Using the original free text responses from the survey in German, S.M. preformed conventional content analysis with the assistance of MAXQDA [23]. Initial themes were labelled using a process of open coding, focusing on themes common across participants as well as those unique to individuals that may offer insight into differences in perspectives. Two other investigators (H.K and D.S.) reviewed the initial analysis to clarify and refine codes, and conversations among the investigators continued until coding differences were resolved and consensus was achieved. This resulted in the initial spectrum of factors influencing people’s willingness to rate their physicians on PRWs. H.K. then analysed the original interview transcriptions in German using conventional content analysis with the assistance of MAXQDA. The initial spectrum of reasons identified during the analysis of the survey responses was modified or added to where needed. The validity of the final spectrum was reviewed by the other investigators (S.M., D.S.) to check consistency and validity. Example quotes were translated into English by S.M.

## Results

Participants identified a total of 21 distinct incentives and disincentives for rating physicians on PRWs, which could be further categorised under the headings: user-specific, PRW-specific and physician-specific. Participants, regardless of whether they had previously rated a physician on a PRW or not, fundamentally agreed on the factors that influenced their willingness to rate physicians on PRWs.

### Incentives for rating a physician

Participants identified a total of 7 different factors that would encourage them to rate a physician (see Table 1 for example quotes).

**Table 1:** Incentive for rating physicians

Type of Factor	Code	Example Quote
User Specific	1. Very positive or very negative experience	“So I think that would be the two extremes. If I was totally delighted, if I had been to a physician and thought, wow, I never experienced that. They were so friendly, great physician, then I could imagine [rating the physician], or if I experienced the exact opposite and thought that wasn’t appropriate at all.” I09
	2. Confidence in anonymity of the evaluators	“Yes of course. Obviously, I would place a lot of value on any kind of anonymization.” I16
	3. Responsibility to provide feedback for the benefit of others	“Well, obviously, I contribute to this. Because I find it important that information is shared.” I02
PRWs Specific	4. Clearly defined criteria for evaluation	“Because you know, if I get scientifically prepared survey, I have the impression that they know what they want and then I can rate.” I12
	5. Rating process simple and fast	“It has to work fast and easy. And there must not be too many questions.” I03
	6. Ability to provide rating via mobile apps	“As I said regarding the low threshold, just for an example, that it is included in another app. Yes, it would be the best, if I had it on my mobile phone, but most websites are still fixed so that you can only see it on a huge computer screen, but my generation are rather mobile-app-users.” I10
Physician Specific	7. Pro-active request to provide feedback	“If the physician said, there is a physician rating website and I would like to ask you leave your opinion there. Then I would probably do that if I had time.” I11

#### User-specific

Participants reported that they would typically only consider rating a physician if they had had a very positive or very negative experience. Participants also place great value in their anonymity and were more likely to rate a physicians if they were confident that their identity would not be revealed. Finally, participants willingness to rate physicians was higher if they felt a responsibility to give feedback to contribute to the improvement of care.

#### PRWs-specific

Participants highlighted the importance of PRWs having clearly defined criteria for evaluation so evaluators understand what is wanted. Participants also stressed that the rating process needed to be simple and fast. Additionally, some participants reported that the ability to rate a physician on a mobile app would have a positive effect on their willingness to rate.

#### Physician specific

Participants reported that pro-active requests from physicians for ratings would increase their willingness to rate.

### Disincentives for rating a physician

Participants identified 14 different factors that would discourage them from rating a physician (see Table 2 for example quotes).

**Table 2:** Disincentives for rating physicians

Type of Factor	Code	Example Quote
User Specific	1. Satisfied with physician	“I was always satisfied” ID 1034
	2. Others have already reported the same opinion	“Others have written that share my opinion” ID 3050
	3. Visiting a physician is an intimate event	“In addition, I feel that visiting a physician is more intimate than visiting a restaurant, which is why I do not want to share any background/concerns/problems”. ID 3394
	4. Subjectivity of the evaluation/physician-patient relationship	“In addition, the question arises: can one justify giving 5 evaluation points to a human relationship? The topic is too complex - in contrast to technical products, hotels, etc.” ID 1018
	5. Fear of negative consequences	“Yes, I think one factor is...fear of negative consequences, even if they can express themselves anonymously, maybe it’s somehow traceable, I don’t know. So I believe that is still a problem. Just like it is in hospitals often, people don’t complain if something goes wrong because they are afraid that they will next treated even worse next time.” I09
	6. Fear to doing harm to the physician	“Yes, because I believe it would be easier for me to give a good rating than a bad one, because I’d have in my mind, that you can destroy quite a lot and because of that I think you have to careful how you write it.” I05
	7. Fear that good physicians will be crowded	“Yes, and if we said that now, I would do that too, but you always have to consider one thing….it is already so full and no available appointments, it would not be better if even more people would go there.” I04
	8. Internet is not the right place voicing criticism	“It is peculiar, physicians are people and I wouldn’t rate my friends on the internet, but I also don’t belong to the generation of social-media. And I think a physician is a person and if one is dissatisfied they should leave immediately, in best case, also tell him [why].” I11
	9. Not qualified to rate aspects of medical practice	“From medical viewpoint, I cannot evaluate medical practitioners on many things.” ID 1223
PRWs Specific	10. Time required too much	“The time required to register on the websites.” ID 2357
	11. Websites too complicated/badly designed	“The rating websites are often cumbersome and often not self-explanatory!” ID 3020
	12. Lack of ratings guidance	“There is no information available about the permitted contents “What and how can be evaluated?”” ID 4144
	13. Mistrust in PRWs operators	“In addition, the operators are often not trustworthy” ID 3193
	14. Lack of consolidation of the PRW market	“There is no market-dominating website, with many ratings for one physician. It is thus not possible, like it is for example with “holiday-check”, to read a kind of “cut-set”.” ID 1296

#### User-specific

Participants identified a wide range of factors that lead them to be less willing to rate a physician. A key overarching theme to emerge concerned the nature of the physician-patient relationship and the potential impact of a rating on that relationship. Participants saw no need to rate their physician if they were satisfied with their physician, or if others had already reported the same opinion. A number of participants also described the nature of the physician-patient relationship as very intimate (particularly compared to visiting a restaurant or hotel) and felt that it was inappropriate to publicly report on this. Furthermore, many participants noted the subjective nature of the physician-patient relationship and of any evaluation of that relationship. While most participants found PRWs helpful as a rough guide, they thought it was more important to get a personal impression of the physician and were reluctant to rate people, as they thought that human relationships were too complex for a rating scale, in contrast to other consumer products and services. In addition, participants expressed fears regarding the impact that rating a physician on PRWs could have, including personal negative consequences, doing harm to the physician, and that good physicians would become crowded.

Participants also raised some more fundamental reasons why they did not rate physicians. At the most basic level, many participants did not think that they are qualified to rate aspects of medical practice. Others held the belief that the internet was simply the wrong place to be voicing criticism, and thought it was more appropriate to raise concerns with the physician directly or the next appropriate level.

#### PRWs-specific

Participants identified several technical barriers to rating a physician, including the time required regarding the registration process, PRWs being too complicated and badly designed, and a lack of rating guidance in terms of allowable content. Furthermore, some participants expressed distrust in the operators of the PRWs, while others commented on the lack of consolidation of the PRWs market, particularly noting that there are too many different PRWs.

### Overarching themes

Two key overarching groups of factors that influenced participants’ willingness to rate physicians on PRWs emerged: (1) factors concerning the physician-patient relationship, and (2) factors regarding technical aspects of PRWs. These themes emerged both in incentives for rating physicians on PRWs (e.g. proactive requests from physicians for rating and the rating process needed to be simple and fast with clear criteria for evaluation), and also disincentives for rating (e.g. the potential impact of a rating on the physician-patient relationship and PRWs being too complicated and badly designed).

## Discussion

As far as we are aware, this is one of the first studies to directly examine factors influencing people’s willingness to rate their physician on PRWs. The two overarching groups of factors identified by this study suggest where future efforts regarding this issue may need to focus.

### Relationship factors

Factors concerning the physician-patient relationship appear to be some of the most important influencing people’s willingness to rate their physician on PRWs, but also the most difficult to address.

Trust is important in all social relationships, although it is potentially even more important in the physician-patient relationship given the inherent imbalances of power, knowledge, and vulnerability that exist [24]. Our study has confirmed the suggestion that patients’ willingness to disclose information about such a relationship is likely to be extremely low unless their expectations are far exceeded or feel that their trust has been violated in some way [14]. Participants repeatedly reported that a very positive or very negative experience in the health care relationship as a crucial precondition for them too be willing to rate a physician. However, while such a rating trend is seen in the online rating of products [25], this is not consistent with the evaluation tendencies that have been found on PRWs. Previous studies in Germany and the United States have consistently found that ratings on PRWs are overwhelmingly positive [6, 26–29]. Participants in our study reported fears regarding the impact a negative rating may have for both themselves and physicians, and such fears have been reported by participants in previous research as well [17]. These fears may be leading patients to self-censor and be one explanation for the low number of negative reviews.

Participants, however, reported that pro-active requests from physicians for ratings would increase their willingness to rate. Such pro-active requests for feedback (positive or negative) within a trusting physician-patient relationship maybe help reduce patients’ fears around posting reviews on PRWs and help increase the number of ratings on PRWs. Unfortunately, however, there is evidence that some physicians are currently urging patients not to post negative reviews on PRWs and are taking legal action against negative reviews that are posted [5]. It has therefore been previously suggested that “the medical profession itself should do more to ensure that patients are not being actively discouraged by physicians to post critical reviews, as they are a potentially important opportunity for physicians to learn and improve care” [14].

### Technical facto**rs**

Participants also reported that there are currently several technical barriers to rating physicians on PRWs, including the time required for registration on PRWs, PRWs being too complicated and badly designed, and a lack of rating guidance in terms of allowable content. These findings support previously research that has highlighted the need to improve the design of PRWs [20]. A recently published study examined the choice-making processes of participants` using the rating website TripAdvisor to select a hotel and the PRW Jameda to select a physician [20]. It was concluded that whereas the information provided on commercial rating websites seems to fit customers’ needs, the similarly designed PRWs did not. It was noted that PRWs are currently set up in the same manner as an “experience good/service”, which consumers can only assess the quality after it has been experienced, by combining general information (location, accessibility, qualifications) of the physician with impersonal anonymous reviews by former patients [20]. However, the selection of a physician could primarily be classified as “credence good/service”, which consumers cannot assess the quality of the product even after consumption and must rely more on interpersonal recommendations than public non-customized information. It was therefore concluded that there is a need for web designers and researchers to consider how PRWs could best provide trustworthy interpersonal information that is adjusted to individuals needs [20].

Furthermore, patients’ perceived inability to evaluate certain aspects of physicians` practice, also suggests that changes to the way data is presented on PRWs may be required. In order to identify the aspects PRWs should offer for evaluation, a recent study by Rothenfluh et al. examined what physicians and patients thought were the relevant factors for identifying a good doctor and whether patients are capable of evaluating these aspects [21]. It was found that physicians and patients agreed that infrastructure, staff, organization, and interpersonal skills are both important aspects of a good physician and can be evaluated by patients. However, while technical skills of a physician and outcomes of care were judged to be the most important aspects of a good physician, both physicians and patients agreed that these aspects could not be evaluable by patients [21]. While combining patient reviews with quality reported has been previously suggested [13], attempts to do so in experiments did not result in better physician selection results [30]. It has therefore been recommended that there is a need for further research to find:

“…PRW formats in which health care consumers can voice their opinion on aspects that are deemed assessable, while condensing and summarizing technical quality of care information in a format that is understandable by health care consumers…” [21].

While some participants reported that the ability to rate a physician on a mobile app would have a positive effect on their willingness to rate, it appears that these participants were not aware that the majority of German PRWs have already had mobile apps for many years. This may indicate that while awareness of PRWs is no longer a key barrier to using PRWs [18], there may not be sufficient awareness of PRWs mobile apps which is acting as an obstacle to some younger people to rate their physicians. PRWs having targeted advertising of these mobile apps to younger people may therefore help efforts to increase physician ratings.

More generally, participants reported that the perceived high number of PRWs in the market was confusing and a disincentive for rating physicians. This supports recent research which examined the development of the frequency of ratings and evaluation tendencies on German PRWs over a 4 year period [14]. It was found that many German PRWs added very few new ratings during this time and that current ratings are spread out across PRWs in an uneven manner [14]. There are, however, signs that the German PRWs market is starting to consolidate. Three major public health insurance companies in Germany (Allgemeine Ortskrankenkasse (AOK), Techniker Krankenkasse (TK) and BARMER GEK), are now all utilising a central database known as “Weisse Liste”; recruiting ratings from their insurees via their own platforms, but pooling these ratings on the shared Weisse Liste. Indeed, the examination of German PRWs found that AOK, Germany’s largest public health insurer, has been able to quickly establish the PRW AOK-Arztnavigator as one of the most used German PRWs since being introduced nationwide in May 2011 (TK and BARMER GEK were not included in the study) [14]. It remains to be seen whether this central database will lead to the German PRWs market further consolidating, however, it does appear to be a positive development and one that may help address concerns reported by participants in this study about the trustworthiness of PRWs operators, as public health insurance companies are seen as the most trustworthy organizations in Germany when it comes to data security [31].

It may also be illuminating to consider previous research on consumers’ willingness to post electronic word of mouth on online rating websites in general. In 2012, Cheung and Lee developed a research model to explain why consumers are willing to spread positive electronic word of mouth on online rating websites, which they then examined using a sample of users from a restaurant rating website in Hong Kong [32]. It was found that three factors were crucial to encourage consumers to share their experience with others on rating websites [32]. Firstly, a sense of belonging (affective commitment) was found to have the biggest impact on consumers’ electronic word of mouth intentions. Second, enjoyment of helping other community members with their decisions (and saving them from having negative experiences) was also found to be crucial in affecting consumers’ electronic word of mouth intentions. Finally, reputation was found to be marginally significant, with some consumers willing to contribute their experiences because they want to be viewed as an expert by others. Reciprocity, moral obligation and knowledge self-efficacy were not found to have a significant relationship with consumers’ electronic word of mouth intentions [32]. These findings suggest further measures that could be taken to improve technical aspects of PRWs and increase the number of ratings. To enhance patients´ sense of belonging to a community, PRWs could create mechanisms that allow patients to (1) create their own (anonymous) profiles, (2) create “groups” for a certain illness or disease, (3) and the ability to communicate directly with other users. The ability to communicate directly with other user may also help address the need to provide trustworthy interpersonal information. In Germany, public health insurance companies may be best placed to achieve a sense of belonging for their PRWs users, with patients likely already having some form of loyalty to the health insurance company. To promote the enjoyment of helping others, PRWs could create a mechanism that allow users who have provided useful reviews to other PRWs users to be identified and informed that they have helped others [32]. Such a mechanism could also include publically visible metrics to contribute to users gaining a positive reputation on the PRW.

### Limitations

This was a qualitative study that did not aim at collecting statistically representative data. Responder bias may have influenced the results; however, as those who responded to our survey and were willing to be interviewed are likely to be generally more interested in the issue, the identified factors influencing these participants´ willingness to rate physicians should be taken seriously. Additionally, all participants came from northern Germany which could have led to a bias as differences may exist between other regions in Germany with respect to PRWs. However, the study used a random sample of an average population from four North German cities of different sizes. Furthermore, non-responder analysis of the survey [18], found no significant difference in gender, and while responders were slightly older than non-responders on average, the effect size was small. We therefore do not think that this issue has significantly impact our results and think the results reveal a more generalizable view of the average population regarding PRWs compared to previous research on PRWs using panel data. Responses were self-reported and, therefore, we do not know the actual use of PRWs.

## Conclusion

Patients clearly value the patient experience data that PRWs make publically available [33]. However, without higher number of ratings, PRWs will continue to have limited value. This study has identified two main overarching groups of factors that can influence patients´ willingness to rate physicians on PRWs. In relation to technical aspects of PRWs, there is a need to improve the design of PRWs to allow for more trustworthy interpersonal information and combining both patient reviews on aspects of physicians´ care that they can evaluate with summaries of technical quality information in an accessible format. Targeted advertising of PRW mobile apps and further consolidation of the PRW market may also help efforts to increase physician ratings. Regarding physician-patient relationship aspects, pro-active requests for feedback (positive or negative) from physicians within a trusting physician-patient relationship may help reduce patients’ fears around posting reviews on PRWs and help increase the number of ratings on PRWs. However, the medical profession needs to do more to ensure that patients are not being actively discouraged by physicians from rating.
